# Unwanted Teenage Pregnancy and Its Complications: A Narrative Review

**DOI:** 10.7759/cureus.32662

**Published:** 2022-12-18

**Authors:** Swarupa Chakole, Shivani Akre, Kapil Sharma, Praful Wasnik, Mayur B Wanjari

**Affiliations:** 1 Department of Community Medicine, Jawaharlal Nehru Medical College, Datta Meghe Institute of Medical Sciences (Deemed to be University), Wardha, IND; 2 Department of Medicine, Jawaharlal Nehru Medical College, Datta Meghe Institute of Medical Sciences (Deemed to be University), Wardha, IND; 3 Department of Research and Development, Jawaharlal Nehru Medical College, Datta Meghe Institute of Medical Sciences (Deemed to be University), Wardha, IND

**Keywords:** teenage, low birth weight, anemia, antenatal care, teenage pregnancy

## Abstract

Teenage pregnancy may have a huge negative social and medical influence on mothers' and children's health globally. Compared to adult primigravida, young women experience more harmful perinatal problems, such as premature births, neonatal fatalities and stillbirths, and the delivery of children with low birth weight. With negative perinatal outcomes, in India, teenage pregnancy is still a widespread and important public health issue that needs urgent attention. One of the main causes of mortality for females between the ages of 15 and 19 is pregnancy and delivery problems. The health of young women in India is, therefore, seriously threatened by adolescent pregnancies. Neonatal and maternal difficulties are more common when a teen gets pregnant. To bring about change, avert problems, and lower the risk of maternal death, specialized antenatal care (ANC) and health education are crucial. In India, teenage pregnancy is very high. To prevent this, there is a need to focus on teenage education regarding safe sexual practices and pregnancy complications.

## Introduction and background

Teenage pregnancy is a societal issue that affects people all over the world. It has major effects on mother and child health, particularly in underdeveloped nations where health care is not as advanced. Despite favoring raising the legal minimum age for female marriage in India to 18 years old, teenage pregnancies are a significant public health issue in India. According to data from the National Family Health Survey (NFHS)-3, 16% of women between the ages of 15 and 19 had already begun having children in India. The state of Jharkhand (28%), followed by West Bengal (25%) and Bihar (25%), both of which are in eastern India, has the greatest percentage of this group with teenage pregnancies. Over half of young women have anemia, 11.4% are stunted, and nearly 47% have a body mass index of less than 18.5 [1]. Low birth weight (LBW), cephalopelvic disproportion (CPD), anemia, pregnancy-induced hypertension, premature labor, anemia, maternal, perinatal, and neonatal mortality, and damage to the reproductive system are just a few of the serious health risks that young women face when they combine poor nutrition with early childbearing [1]. It brings up a number of human rights problems. For example, a pregnant adolescent girl who is forced to miss school is denied her right to an education. She also denied her right to health because she is not allowed to use any kind of contraception or access knowledge on reproductive health. Many teenagers are not physically or mentally prepared for pregnancy or delivery, which increases the risk of problems and even life-threatening health effects. Early school leaving and health issues compromise their ability to make money [2].

## Review

Causes of teenage pregnancy

Teenage pregnancy is a common problem that is more likely to affect vulnerable populations due to factors including poverty, illiteracy, and a lack of job prospects. It continues to be a significant factor in infant mortality and maternal mortality, as well as intergenerational cycles of illness and poverty. Teenage pregnancy incidences were shown to be mostly caused by a lack of education, lack of access to contraception and health information, and autonomy in making decisions. Teenage pregnancy is greatly influenced by early marriage, rape, or sexual abuse of married or unmarried females. Unwanted births and the spread of sexually transmitted infections (STIs) are both facilitated by the partner's refusal or resistance to use any kind of contraception [3].

Effects of teenage pregnancy

For a young mother and her child, life may be challenging. Teenage moms are more likely to leave school for childcare compared to other females. Due to her little schooling, a teen mother may lack the skills necessary for work, making it challenging for her to find and maintain a career and establish her source of income [4]. Young mothers who have children may become financially dependent on their families or government support. The aforementioned factors make teen mothers more likely to be poor. Whatever the circumstances, becoming pregnant is a challenge for every woman. The crisis, however, is far more intense for the teenager since it adds yet another degree of complexity to a physically and emotionally stressful time. Due to financial strain, societal stigmas, or a lack of support from her family and community, the young mother may also experience mental health problems, including stress, sadness, and suicidal thoughts. They encounter loneliness, guilt that causes stress and despair, poor self-esteem that causes them to lose interest in their studies, few career opportunities, and a lack of a support system [4,5].

Medical consequences of teenage pregnancy

Pregnant teenagers are less likely to obtain prenatal care because they may not be aware of their pregnancy or may not know enough about it to seek it out before the third trimester. Teenage moms experience more preterm deliveries and LBW globally as a result of inadequate prenatal care [4]. Girls under the age of 14 are more at risk for medical issues since an undeveloped pelvis might make delivery more challenging. Young women under the age of 20 are more likely to experience obstructed labor, which, in the absence of a cesarean section, can result in an obstetric fistula, a break in the birth canal that allows urine and/or excrement to flow out. In underdeveloped nations, the leading causes of death for girls between the ages of 15 and 19 are pregnancy and childbirth-related complications [4].

Inadequate eating practices typical in adolescence, such as efforts to lose weight through diets and meal skipping, snacking, and fast-food intake, leave many pregnant teenagers vulnerable to nutritional inadequacies. Anemia is mostly caused by a poor diet and inadequate prenatal care. Young moms are at increased risk of preterm birth, LBW infants, preeclampsia, eclampsia, premature membrane rupture, gestational diabetes mellitus, pregnancy-induced hypertension, urinary infections, and hemorrhagic syndromes [5,6].

The preterm birth of newborns also has life-threatening morbidities and mortalities, which include intrauterine growth restriction (IUGR), necrotizing enterocolitis, hyaline membrane disease, alveolar proteinosis in newborns, respiratory distress syndrome, bronchopulmonary dysplasia, and retinopathy of prematurity. Improper intake of folic acid may cause a baby to be born with neural tube defects or other comorbid syndromes and congenital defects. Birth injuries may also occur when instrumental delivery is done [6]. In comparison with the 6.97% rate in the general population, the combined proportion of spontaneous abortions and stillbirths is 9.84%, which is relatively high in teenage pregnancies. Poor nutrition, anemia, preeclampsia, and a high incidence of chorioamnionitis caused by sexually transmitted diseases (STDs) and human immunodeficiency virus (HIV) in teen pregnancies are the causes. According to the same study, teenage girls have a medical termination rate (MTP) of 9.15%, compared to 5.07% in the overall population [7]. Teenage girls execute 14% of the estimated 20 million unsafe abortions annually, which result in 68,000 fatalities [4].

**Figure 1:**
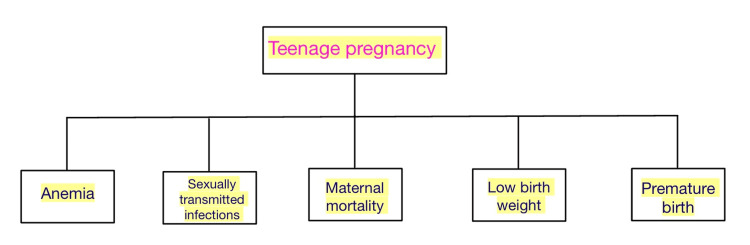
Complications associated with teenage pregnancy. Image credit: Dr. Kapil Sharma.

Teenage pregnancy can result in an inadequate pelvis, obstructed labor, infant mortality, or maternal death. Because the pelvic architecture is not yet fully developed and ready for delivery, CPD is a typical issue during labor in teenage pregnancies. This can further result in longer labor, obstructed labor, and hypotonic uterine contractions (Figure 1) [7,8].

Preeclampsia in teenage pregnancy

Preeclampsia, a pregnancy disorder characterized by increasing hypertension that can affect several organs and have adverse consequences on both the mother and the fetus, is more prevalent in young mothers, particularly those who are primigravid [8,9]. Since an underdeveloped uterus and irregular menstrual cycles might affect decidualization, deep placentation, and spiral artery remodeling, preeclampsia can be connected to these conditions [8]. The only effective therapy for preeclampsia is the delivery of the fetus. Fetal prematurity can be a major issue if preeclampsia develops before 37 weeks of pregnancy. To avoid the major side effects of the illness, preeclamptic women must undergo attentive screening and monitoring of signs and symptoms [10].

Premature rupture of membranes

According to the definition, premature membrane rupture is when the membranes of the sac burst before 37 weeks of pregnancy [11]. Some of the numerous elements that may contribute to the pathophysiology include race and social position, smoking, sexual behavior, malnutrition, vaginal bleeding, and genital tract infections [11]. Girls in adolescence are more prone to infections that result in membrane rupture by raising inflammatory markers such as interleukins and prostaglandins [12]. Adolescent girls had much higher rates of premature membrane rupture, according to a study's findings. Additionally, it should be emphasized that teenage girls had a higher rate of preterm membrane rupture during term births [13]. Membrane rupture can be diagnosed by direct speculum inspection, detecting leaking amniotic fluid, nitrazine test, crystallography, and ultrasound. Premature membrane rupture can be effectively managed with the support of the mother's use of antibiotics, gestational age-appropriate corticosteroid dosage, magnesium sulfate for fetal neuroprotection, tocolytic medications, and the best time and mode of delivery [14].

Anemia in teenage pregnancy

According to the World Health Organization (WHO), anemia is a reduction in the oxygen-carrying capacity of hemoglobin or red blood cells. Hemoglobin levels under 7 g/dL are considered severe anemia during pregnancy, moderate anemia is defined as 7-9.99 g/dL, and mild anemia is defined as levels under 11 g/dL [15,16]. Due to the higher iron requirements during a specific stage of fast growth where significant biological changes are taking place, pregnant teenagers are more likely to experience anemia. This may result in iron deficiency, which might harm both fetuses and teenagers physically and cognitively [17]. The WHO recommends that beginning of pregnancy and continuing for three months after delivery, every pregnant woman should take a preventative supplement containing 40 mg of elemental iron [18].

Teenage sexual activity and the transmission of diseases

Hepatitis B, trichomoniasis, syphilis, gonorrhea, chlamydia, HIV, herpes simplex viruses, and human papillomavirus (HPV) infections are STIs that are frequently spread during teenage sexual contact. Vertical transmission of these STIs can cause injury to the fetus [19,20]. Due to a lack of early sex education, adolescents are noticeably more vulnerable to STIs. Additional elements contributing to the spread of diseases include drug usage, gender, socioeconomic inequalities, and false beliefs among teenagers [21].

Neonatal complications of teenage pregnancy

A frequent issue with pregnancy at a young age is preterm birth. Prematurity is a contributing factor to the physical, mental, behavioral, auditory, visual, and social-emotional development abnormalities as well as respiratory emergencies, immunological diseases, and central nervous system diseases in children [22-25]. Preterm birth is the delivery of a child before 37 weeks of gestation, according to the WHO [26].

Another often-occurring side effect of teenage pregnancy is LBW [27]. LBW is defined as a birth weight of 2,500 g or less at delivery by the WHO [28]. Proper dietary supplements, along with routine, on-time checkups, must be taken into consideration to prevent LBW. During teenage pregnancies, neonatal mortality is higher [29,30]. Infant fatalities that occur within the first 28 days of life are referred to as "neonatal mortality" [30]. Mothers and infants should get extra care and nutrition in order to lower neonatal mortality.

Teenage pregnancy prevention and care

Unwanted pregnancy at a young age, in particular, causes the female great emotional and mental stress. Consequently, programs that are effective at postponing efforts at sexual activity are the first and primary lines of defense. Talking openly, honestly, and in an informative manner with preteens and teenagers is needed between parents, schools, social workers, and medical experts. Teenagers might also receive guidance from them on how to avoid unintended pregnancies. While some therapies have focused just on education and others on encouraging adolescents to refrain from sexual activity, others have emphasized training teenagers' social and cognitive abilities that are considered to lower the risk of early pregnancy [4,30]. The government needs to consider mandating sex and relationship education in both elementary and secondary schools. Clinics with ties to local colleges can lower pregnancy rates [4,30]. Some crucial preventive measures include preventing pre-adult marriage, increasing comprehension and assistance for restricting conception before the age of 20, encouraging teens to use contraceptives more often, minimizing forced sexual activity in teens, and reducing unsafe abortions that are hazardous [30].

## Conclusions

Complication rates for pregnancies in young women are much higher. This might hinder their growth and development, rob them of their childhood and education, and worsen the nation's general health in the process. This is the right time to concentrate on this issue. Transforming today's teenage girls into healthy, responsible adults who will produce a healthy new generation will undoubtedly be aided by education, dietary guidance, and family planning, as well as increasing community awareness and explaining to schoolgirls the significance of postponing marriage, family life, reproductive health, and population education. In conclusion, it is important for all parties, parents, educators, social workers, the government, and individuals, to do their bit to stop adolescent pregnancy from developing into a major issue that might result in death or becoming one that is brought up and debated in the legislature. In addition, the community must play a part in resolving the situation and avoiding it from worsening, particularly for the unmarried couple dealing with an unintended pregnancy. However, the problem of unintended pregnancy among unmarried couples may be prevented with the right supervision and counseling. On the other side, patiently waiting to reach marriageable age and making marriage plans with family and friends would undoubtedly result in a happy and healthy child or children in a family. There is a need for sex education programs to be offered in schools that will educate students accurately about STIs and contraception for pregnancy.
